# [*N*,*N*′-Bis(2,6-diisopropyl­phen­yl)methanimidamidato][η^8^-1,4-bis­(tri­methyl­sil­yl)cyclo­octa­tetra­enyl](tetra­hydro­furan)­samarium(III) toluene monosolvate

**DOI:** 10.1107/S1600536810048531

**Published:** 2010-11-27

**Authors:** Anja Edelmann, Cristian G. Hrib, Liane Hilfert, Steffen Blaurock, Frank T. Edelmann

**Affiliations:** a Chemisches Institut der Otto-von-Guericke-Universität, Universitätsplatz 2, D-39106 Magdeburg, Germany

## Abstract

The title compound, [Sm(C_25_H_35_N_2_)(C_14_H_24_Si_2_)(C_4_H_8_O)]·C_7_H_8_, was prepared by treatment of anhydrous samarium trichloride with a 1:1 mixture of *in situ*-prepared Li(DippForm) [DippFormH = *N*,*N*′-bis­(2,6-diisopropyl­phen­yl)methanimidamide] and Li_2_(COT′′) [COT′′ = 1,4-bis­(trimethyl­sil­yl)cyclo­octa­tetra­enyl] in tetra­hydro­furan (THF). Despite the presence of two very bulky ligands (COT′′ and DippForm), the mol­ecule still contains one coordinated THF ligand. The overall coordination geometry around the Sm^III^ atom resembles a three-legged piano-stool with the COT′′ ligand being η^8^-coordinated and the DippForm^−^ anion acting as an *N*,*N*′-chelating ligand [Sm—N = 2.5555 (15) and 2.4699 (15) Å]. The asymmetric unit also contains a disordered mol­ecule of toluene, the refined ratio of the two components being 0.80 (4):0.20 (4).

## Related literature

For review articles on the search for alternative specta­tor ligands other than cyclo­penta­dienyls which are able to satisfy the coordination requirements of the large lanthanide cations, see: Edelmann (1995 ▶, 2009 ▶); Bailey & Pace (2001 ▶); Edelmann *et al.* (2002 ▶). For related complexes, see: Schumann *et al.* (1995 ▶). For bulky formamidinate ligands, see: Cole *et al.* (2007 ▶); Junk & Cole (2007 ▶). For the COT′′ ligand, see: Burton *et al.* (1989 ▶,1993 ▶).
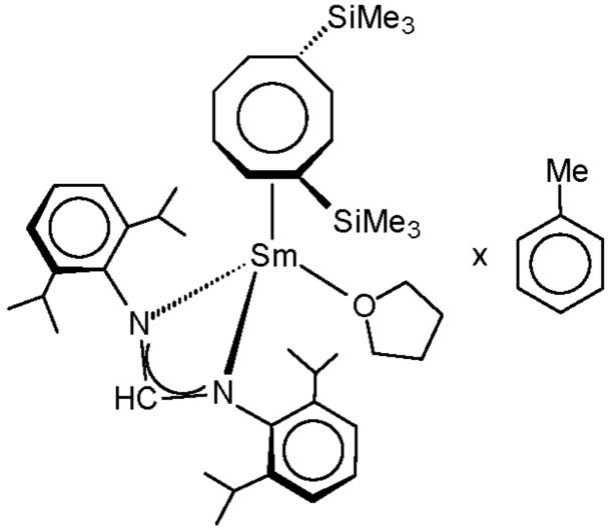


## Experimental

### 

#### Crystal data

[Sm(C_25_H_35_N_2_)(C_14_H_24_Si_2_)(C_4_H_8_O)]·C_7_H_8_
*M* *_r_* = 926.65
Monoclinic, 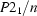

*a* = 18.490 (4) Å
*b* = 11.166 (2) Å
*c* = 24.865 (5) Å
β = 107.80 (3)°
*V* = 4887.9 (19) Å^3^
*Z* = 4
Mo *K*α radiation
μ = 1.29 mm^−1^
*T* = 133 K
0.40 × 0.22 × 0.14 mm

#### Data collection

Stoe IPDS 2T diffractometer
Absorption correction: numerical (*X-SHAPE* and *X-RED32*; Stoe & Cie, 2002 ▶) *T*_min_ = 0.978, *T*_max_ = 0.992
13121 measured reflections
13121 independent reflections
10516 reflections with *I* > 2σ(*I*)

#### Refinement

*R*[*F* ^2^ > 2σ(*F* ^2^)] = 0.024
*wR*(*F* ^2^) = 0.056
*S* = 0.90
13121 reflections
511 parameters
10 restraints
H-atom parameters constrained
Δρ_max_ = 1.35 e Å^−3^
Δρ_min_ = −1.81 e Å^−3^

### 

Data collection: *X-AREA* (Stoe & Cie, 2002 ▶); cell refinement: *X-AREA*; data reduction: *X-RED32* (Stoe & Cie, 2002 ▶); program(s) used to solve structure: *SHELXS97* (Sheldrick, 2008 ▶); program(s) used to refine structure: *SHELXL97* (Sheldrick, 2008 ▶); molecular graphics: *XP* (Siemens, 1994 ▶); software used to prepare material for publication: *SHELXL97*.

## Supplementary Material

Crystal structure: contains datablocks I, global. DOI: 10.1107/S1600536810048531/zs2077sup1.cif

Structure factors: contains datablocks I. DOI: 10.1107/S1600536810048531/zs2077Isup2.hkl

Additional supplementary materials:  crystallographic information; 3D view; checkCIF report

## Figures and Tables

**Fig. 1.**
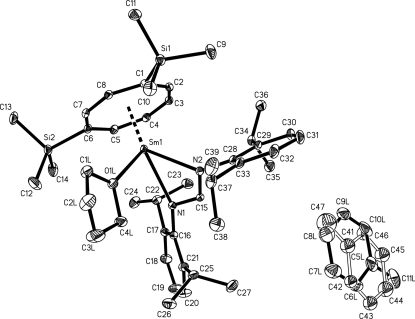
The molecule of the title compound in the crystal. Thermal ellipsoids represent 50% probability levels.

## supplementary crystallographic information

### Comment

A hot topic in current organolanthanide chemistry is the search for alternative spectator ligands other than cyclopentadienyls which are able to satisfy the coordination requirements of the large lanthanide cations (Edelmann *et al.*, 2002). Among the most successful approaches in this field is the use of amidinate ligands of the general type [RC(NR')_2_]^-^ (*R* = H, alkyl, aryl; *R*' = alkyl, cycloalkyl, aryl, SiMe_3_) which can be regarded as steric cyclopentadienyl equivalents (Bailey & Pace, 2001; Edelmann, 2009). Closely related very bulky *N,N'*-disubstituted formamidinate ligands such as DippForm^-^ [DippFormH = *N,N'*-bis(2,6-diisopropylphenyl)methanimidamide] have also been frequently employed in this area (Cole *et al.*, 2007; Junk & Cole, 2007]. Another ligand system besides cyclopentadienyl, which is traditionally very important in organolanthanide chemistry, is the cyclooctatetraenyl dianion (= COT) (Edelmann, 1995) and its ring-substituted derivatives such as 1,4-bis(trimethylsilyl)cyclooctatetraenyl (= COT'') (Burton *et al.*, 1989; Burton *et al.*, 1993). Rare-earth metal complexes of the type (COT)Ln[RC(NR')_2_](THF) have been reported by Schumann *et al.* (1995). These compounds all comprise the unsubstituted COT ligand and moderately bulky *N*,*N*'-bis(trimethylsilyl) benzamidinate ligands (Schumann *et al.*, 1995). We were interested in the question if THF-free compounds of this type would be accessible by combining both the bulky COT'' and the DippForm ligand in the coordination sphere of a lanthanide ion. The first compound of this series, [η^8^-1,4-bis(trimethylsilyl)cyclooctatetraenyl][*N*,*N*'-bis(2,6-diisopropyl&shy;phenyl)formamidinato](tetrahydrofuran)samarium(III) [= (COT'')Sm&shy;(DippForm)(THF), was synthesized by treatment of anhydrous samarium trichloride simultaneously with *in situ*-prepared Li(DippForm) and Li_2_(COT'') in THF. Work-up followed by recrystallization from toluene afforded the dark red title compound. Besides X-ray crystallography, the title compound was also characterized by elemental analysis and spectroscopic methods. Dark red, highly air-sensitive, rod-like single crystals of the title compound were obtained by slow cooling of a saturated solution in toluene to 278 K. Surprisingly, despite the presence of two very bulky ligands, COT'' and DippForm, the molecule still contains one coordinated THF ligand. Thus the overall coordination geometry around Sm resembles a three-legged piano-stool with the COT'' ligand being η^8^-coordinated and the DippForm^-^ anion acting as *N,N'*-chelating ligand (Sm—N distances: Sm1—N1, 2.5555 (15); Sm1—N2, 2.4699 (15) Å). The N1—C15—N2 angle of the formamidinate unit is 120.10 (15)°.

### Experimental

*Preparation of [η^8^-1,4-bis(trimethylsilyl)cyclooctatetraenyl][N,N'-bis(2,6-diisopropyl&shy;phenyl)formamidinato](tetrahydrofuran)samarium(III)*: The reaction was carried out under rigorous exclusion of air and moisture. In a 100 ml-Schlenk-flask, a mixture of 1,4-bis(trimethylsilyl)cycloocta-2,5,7-triene (2.00 g, 8.1 mmol) and *N,N'*-bis(2,6-diisopropylphenyl)methanimidamide (= DippFormH, 2.90 g, 8.1 mmol) were dissolved in THF (25 ml) and a 1.6 N solution of *n*-butyllithium in *n*-hexane (15 ml, 24 mmol) was added. Metalation as completed by stirring for 3 h at room temperature. Anhydrous SmCl_3_ (2.1 g, 8.1 mmol) was added as solid and stirring was continued for 24 h. A white precipitate (LiCl) was removed by filtration, and the clear red.brown flitrate was evaporated to dryness. The residue was extracted with warm (*ca* 323 K) toluene (2x20 ml), the combined axtracts were filtered again and diluted with *n*-pentane (30 ml). Cooling to 278 K for 2–3 d afforded 5.27 g (78%) of [η^8^-1,4-bis(trimethylsilyl)cyclooctatetraenyl][*N,N'*-bis(2,6-diisopropylphenyl)formamidinato](tetrahydrofuran)samarium(III) as a dark red microcrystalline solid. Dark red, rod-like X-ray quality single-crystals of the mono-toluene solvate were obtained by recrystallization from toluene. Anal. calcd for C_43_H_67_N_2_OSi_2_Sm (834.55 g/mol): C 61.89, H 8.09, N 3.36; found: C 62.56, H 8.14, N 3.50%. IR (KBr pellet): *ν*_max_ 2961 (*versus*), 2867 (*m*), 1665 (*s*), 1643 (*m*), 1588 (*m*), 1528 (*versus*), 1456 (*m*), 1438 (*m*), 1383 (w), 1361 (w), 1332 (*m*), 1320 (*m*), 1319 (*s*), 1285 (*s*), 1248 (*versus*), 1190 (*m*), 1160 (*s*), 1099 (*m*), 1046 (*m*), 937 (*m*), 837 (*versus*), 799 (*m*), 754 (*s*) cm^-1. 1^H NMR (400.1 MHz, THF-*d*_8_, 298 K): *d* = 12.99 (s br, 2H), 9.28 (s br, 2H), 8.60 (s br, 2H) (COT'' ring-H); 8.34 (s, 1H, N—C*H*-N); 7.07–7.21 (m, 11H, phenyl ring-H, DippForm + toluene); 3.60 (m, 4H, THF); 2.94 (m, 4H, C*H*(CH_3_)_2_); 2.31 (s, 3H, C_6_H_5_—C*H*_3_); (1.77 (m, 4H, THF); 1.17 (d, 24H, CH(C*H*_3_)_2_); 0.74 (s, 18H, Si(C*H*_3_)_3_) p.p.m.. ^13^C NMR (100.6 MHz, THF-*d*_8_, 298 K): δ = 190.6 (N-*C*H—N), 144.6, 141.9, 124.4, 123.8 (phenyl ring-*C*, DippForm); 138.3, 129.5, 128.8, 125.9 (*C*_6_H_5_—CH_3_); 95.1, 90.3, 84.2, 83.8 (COT'' ring-*C*); 68.1 (THF); 29.8 (CH(*C*H_3_)_2_); 28.1 *C*H(CH_3_)_2_); 26.2 (THF); 21.4 (H_3_*C*-C_6_H_5_); -0.27 (Si(*C*H_3_)_3_) p.p.m.. ^29^Si NMR (79.5 MHz, THF-*d*_8_, 298 K): δ = -54.4 (*Si*Me_3_) p.p.m.. EI—MS: *m/z* 834.2 (18%) [*M*]^+^, 514.9 (100%) [Sm(DippForm)]^+^, 399.7 (3") [Sm(COT'')]^+^, 365.0 (88%) [DippFormH]^+^, 72.9 (16%) [THF]^+^.

### Refinement

The asymmetric unit contains a disordered molecule of toluene. The refined ratio of the two components is 0.80 (4):0.20 (4). Since the refined ratio of the second component is only 20% it was not possible to find the maxima for the hydrogen atoms of the methyl group. Also the carbon atoms of the second component were refined isotropically. The hydrogen atoms were included using a riding model, with aromatic C—H = 0.95 Å, methine C—H = 1.00 Å, methylene C—H = 0.99 Å [*U*_iso_(H) = 1.2*U*eq(C)] and methyl C—H = 0.98 Å [*U*_iso_(H) = 1.5*U*eq(C)].

### Crystal data

**Table:**

[Sm(C_25_H_35_N_2_)(C_14_H_24_Si_2_)(C_4_H_8_O)]·C_7_H_8_	*F*(000) = 1948
*M**_r_* = 926.65	*D*_x_ = 1.259 Mg m^−^^3^
Monoclinic, *P*2_1_/*n*	Mo *K*α radiation, λ = 0.71073 Å
Hall symbol: -P 2yn	Cell parameters from 2203 reflections
*a* = 18.490 (4) Å	θ = 2.0–29.3°
*b* = 11.166 (2) Å	µ = 1.29 mm^−^^1^
*c* = 24.865 (5) Å	*T* = 133 K
β = 107.80 (3)°	Rod, red
*V* = 4887.9 (19) Å^3^	0.40 × 0.22 × 0.14 mm
*Z* = 4	

### Data collection

**Table:**

Stoe IPDS 2T diffractometer	13121 independent reflections
Radiation source: fine-focus sealed tube	10516 reflections with *I* > 2σ(*I*)
graphite	*R*_int_ = 0.0000
ω scans	θ_max_ = 29.3°, θ_min_ = 2.0°
Absorption correction: numerical (*X-SHAPE* and *X-RED32*; Stoe & Cie, 2002)	*h* = −25→24
*T*_min_ = 0.978, *T*_max_ = 0.992	*k* = 0→15
13121 measured reflections	*l* = 0→34

### Refinement

**Table:**

Refinement on *F*^2^	Primary atom site location: structure-invariant direct methods
Least-squares matrix: full	Secondary atom site location: difference Fourier map
*R*[*F*^2^ > 2σ(*F*^2^)] = 0.024	Hydrogen site location: inferred from neighbouring sites
*wR*(*F*^2^) = 0.056	H-atom parameters constrained
*S* = 0.90	*w* = 1/[σ^2^(*F*_o_^2^) + (0.0371*P*)^2^] where *P* = (*F*_o_^2^ + 2*F*_c_^2^)/3
13121 reflections	(Δ/σ)_max_ = 0.003
511 parameters	Δρ_max_ = 1.35 e Å^−^^3^
10 restraints	Δρ_min_ = −1.80 e Å^−^^3^

### Special details

**Table:**

Geometry. All e.s.d.'s (except the e.s.d. in the dihedral angle between two l.s. planes) are estimated using the full covariance matrix. The cell e.s.d.'s are taken into account individually in the estimation of e.s.d.'s in distances, angles and torsion angles; correlations between e.s.d.'s in cell parameters are only used when they are defined by crystal symmetry. An approximate (isotropic) treatment of cell e.s.d.'s is used for estimating e.s.d.'s involving l.s. planes.
Refinement. Refinement of *F*^2^ against ALL reflections. The weighted *R*-factor *wR* and goodness of fit *S* are based on *F*^2^, conventional *R*-factors *R* are based on *F*, with *F* set to zero for negative *F*^2^. The threshold expression of *F*^2^ > σ(*F*^2^) is used only for calculating *R*-factors(gt) *etc*. and is not relevant to the choice of reflections for refinement. *R*-factors based on *F*^2^ are statistically about twice as large as those based on *F*, and *R*- factors based on ALL data will be even larger. The asymmetric unit contains a disordered molecule of toluene. The refined ratio of the two components is 0.80 (4):0.20 (4). Since the refined ratio of the second component is only 20% it was not possible to find the maxima for the hydrogen atoms of the methyl group. Also the carbon atoms of the second component were refined isotropically.

### Fractional atomic coordinates and isotropic or equivalent isotropic displacement parameters (Å^2^)

**Table:**

	*x*	*y*	*z*	*U*_iso_*/*U*_eq_	Occ. (<1)
Sm1	0.405603 (5)	0.266319 (7)	0.245665 (4)	0.01538 (3)	
Si1	0.21411 (3)	0.30376 (5)	0.10579 (2)	0.02413 (11)	
Si2	0.35521 (3)	0.30346 (5)	0.39783 (2)	0.02455 (11)	
N1	0.55006 (8)	0.26252 (14)	0.26855 (6)	0.0187 (3)	
N2	0.47622 (8)	0.28076 (14)	0.17613 (6)	0.0189 (3)	
O1L	0.42110 (8)	0.48577 (12)	0.25701 (6)	0.0266 (3)	
C1L	0.36356 (13)	0.58031 (18)	0.24756 (11)	0.0330 (5)	
H1LA	0.3459	0.5901	0.2811	0.040*	
H1LB	0.3193	0.5614	0.2145	0.040*	
C2L	0.40251 (16)	0.6908 (2)	0.23726 (15)	0.0518 (7)	
H2LA	0.3985	0.6987	0.1968	0.062*	
H2LB	0.3806	0.7631	0.2494	0.062*	
C3L	0.48233 (17)	0.6729 (2)	0.27211 (19)	0.0707 (11)	
H3LA	0.4898	0.6971	0.3118	0.085*	
H3LB	0.5173	0.7198	0.2570	0.085*	
C4L	0.49522 (13)	0.5411 (2)	0.26807 (11)	0.0352 (5)	
H4LA	0.5168	0.5240	0.2370	0.042*	
H4LB	0.5307	0.5107	0.3038	0.042*	
C1	0.26614 (9)	0.23910 (16)	0.17715 (7)	0.0194 (3)	
C2	0.30412 (10)	0.12855 (16)	0.17700 (8)	0.0199 (3)	
H2	0.2986	0.1012	0.1398	0.024*	
C3	0.34796 (10)	0.04931 (16)	0.21837 (8)	0.0199 (3)	
H3	0.3616	−0.0211	0.2023	0.024*	
C4	0.37646 (10)	0.04961 (16)	0.27783 (8)	0.0198 (3)	
H4	0.4047	−0.0209	0.2920	0.024*	
C5	0.37376 (10)	0.12785 (17)	0.32168 (8)	0.0208 (3)	
H5	0.4034	0.0996	0.3577	0.025*	
C6	0.33760 (10)	0.23866 (17)	0.32523 (7)	0.0203 (3)	
C7	0.28865 (10)	0.31161 (17)	0.28213 (8)	0.0200 (3)	
H7	0.2704	0.3783	0.2979	0.024*	
C8	0.25975 (10)	0.31212 (17)	0.22249 (8)	0.0192 (3)	
H8	0.2275	0.3790	0.2091	0.023*	
C9	0.23547 (15)	0.2177 (2)	0.04801 (9)	0.0405 (5)	
H9A	0.2904	0.2183	0.0539	0.061*	
H9B	0.2092	0.2547	0.0115	0.061*	
H9C	0.2181	0.1348	0.0483	0.061*	
C10	0.24198 (14)	0.4632 (2)	0.10097 (10)	0.0367 (5)	
H10A	0.2967	0.4676	0.1062	0.055*	
H10B	0.2300	0.5102	0.1304	0.055*	
H10C	0.2140	0.4953	0.0638	0.055*	
C11	0.10940 (12)	0.3030 (2)	0.09541 (10)	0.0344 (5)	
H11A	0.0825	0.3366	0.0583	0.052*	
H11B	0.0986	0.3513	0.1249	0.052*	
H11C	0.0923	0.2205	0.0976	0.052*	
C12	0.39430 (18)	0.4586 (3)	0.40028 (11)	0.0494 (7)	
H12A	0.4034	0.4919	0.4382	0.074*	
H12B	0.3578	0.5091	0.3727	0.074*	
H12C	0.4422	0.4561	0.3911	0.074*	
C13	0.26264 (13)	0.3122 (2)	0.41321 (10)	0.0367 (5)	
H13A	0.2413	0.2316	0.4122	0.055*	
H13B	0.2273	0.3626	0.3848	0.055*	
H13C	0.2707	0.3471	0.4508	0.055*	
C14	0.42327 (15)	0.2115 (3)	0.45355 (10)	0.0460 (6)	
H14A	0.4036	0.1298	0.4526	0.069*	
H14B	0.4293	0.2471	0.4907	0.069*	
H14C	0.4726	0.2095	0.4465	0.069*	
C15	0.54287 (9)	0.25826 (16)	0.21399 (7)	0.0185 (3)	
H15	0.5855	0.2391	0.2019	0.022*	
C16	0.62515 (10)	0.25293 (16)	0.30688 (7)	0.0204 (3)	
C17	0.63883 (10)	0.16724 (17)	0.35098 (8)	0.0225 (4)	
C18	0.71151 (12)	0.1569 (2)	0.38879 (10)	0.0324 (4)	
H18A	0.7207	0.1003	0.4187	0.039*	
C19	0.77072 (13)	0.2270 (2)	0.38395 (11)	0.0426 (6)	
H19A	0.8203	0.2175	0.4097	0.051*	
C20	0.75717 (12)	0.3110 (2)	0.34141 (11)	0.0382 (5)	
H20A	0.7981	0.3593	0.3384	0.046*	
C21	0.68516 (11)	0.32726 (18)	0.30256 (9)	0.0261 (4)	
C22	0.57698 (11)	0.08184 (18)	0.35439 (8)	0.0247 (4)	
H22A	0.5274	0.1247	0.3395	0.030*	
C23	0.57511 (13)	−0.0257 (2)	0.31580 (10)	0.0353 (5)	
H23A	0.5706	0.0027	0.2776	0.053*	
H23B	0.5315	−0.0765	0.3147	0.053*	
H23C	0.6221	−0.0720	0.3304	0.053*	
C24	0.58400 (13)	0.0399 (2)	0.41440 (10)	0.0365 (5)	
H24A	0.5851	0.1096	0.4385	0.055*	
H24B	0.6310	−0.0062	0.4294	0.055*	
H24C	0.5404	−0.0107	0.4136	0.055*	
C25	0.67652 (11)	0.42579 (19)	0.25866 (9)	0.0272 (4)	
H25A	0.6218	0.4287	0.2355	0.033*	
C26	0.69770 (14)	0.5494 (2)	0.28573 (10)	0.0376 (5)	
H26A	0.6676	0.5658	0.3112	0.056*	
H26B	0.6872	0.6107	0.2561	0.056*	
H26C	0.7519	0.5507	0.3071	0.056*	
C27	0.72306 (14)	0.3984 (2)	0.21843 (11)	0.0411 (6)	
H27A	0.7088	0.3194	0.2012	0.062*	
H27B	0.7773	0.3987	0.2396	0.062*	
H27C	0.7128	0.4595	0.1888	0.062*	
C28	0.46910 (9)	0.25978 (18)	0.11824 (7)	0.0208 (3)	
C29	0.47324 (10)	0.14315 (19)	0.09802 (8)	0.0234 (4)	
C30	0.46624 (12)	0.1284 (2)	0.04083 (9)	0.0334 (5)	
H30A	0.4694	0.0501	0.0267	0.040*	
C31	0.45484 (13)	0.2243 (3)	0.00446 (9)	0.0396 (5)	
H31A	0.4511	0.2123	−0.0341	0.047*	
C32	0.44880 (13)	0.3383 (2)	0.02441 (9)	0.0352 (5)	
H32A	0.4396	0.4040	−0.0010	0.042*	
C33	0.45594 (11)	0.3585 (2)	0.08093 (8)	0.0259 (4)	
C34	0.48399 (11)	0.03259 (18)	0.13561 (9)	0.0263 (4)	
H34A	0.4747	0.0567	0.1717	0.032*	
C35	0.56497 (13)	−0.0169 (2)	0.15032 (10)	0.0348 (5)	
H35A	0.6013	0.0464	0.1677	0.052*	
H35B	0.5746	−0.0451	0.1158	0.052*	
H35C	0.5708	−0.0838	0.1768	0.052*	
C36	0.42773 (14)	−0.0665 (2)	0.10883 (11)	0.0388 (5)	
H36A	0.3758	−0.0352	0.0993	0.058*	
H36B	0.4337	−0.1330	0.1356	0.058*	
H36C	0.4375	−0.0950	0.0744	0.058*	
C37	0.45387 (13)	0.4857 (2)	0.10222 (9)	0.0319 (5)	
H37A	0.4339	0.4823	0.1353	0.038*	
C38	0.53453 (15)	0.5380 (2)	0.12273 (11)	0.0441 (6)	
H38A	0.5675	0.4841	0.1508	0.066*	
H38B	0.5332	0.6166	0.1399	0.066*	
H38C	0.5543	0.5465	0.0906	0.066*	
C39	0.40268 (16)	0.5706 (2)	0.05834 (11)	0.0449 (6)	
H39A	0.3512	0.5375	0.0449	0.067*	
H39B	0.4229	0.5795	0.0264	0.067*	
H39C	0.4013	0.6491	0.0757	0.067*	
C5L	0.81056 (15)	0.0907 (2)	0.06760 (10)	0.0519 (10)	0.800 (5)
C6L	0.81808 (14)	0.2116 (2)	0.08157 (11)	0.0462 (9)	0.800 (5)
H6L	0.8667	0.2485	0.0914	0.055*	0.800 (5)
C7L	0.75450 (18)	0.27851 (18)	0.08111 (12)	0.0606 (11)	0.800 (5)
H7L	0.7596	0.3611	0.0907	0.073*	0.800 (5)
C8L	0.68339 (14)	0.2245 (3)	0.06669 (12)	0.0651 (14)	0.800 (5)
H8L	0.6399	0.2702	0.0664	0.078*	0.800 (5)
C9L	0.67587 (14)	0.1036 (3)	0.05272 (12)	0.0628 (13)	0.800 (5)
H9L	0.6273	0.0667	0.0429	0.075*	0.800 (5)
C10L	0.73945 (18)	0.03674 (19)	0.05318 (11)	0.069 (2)	0.800 (5)
H10L	0.7343	−0.0459	0.0436	0.083*	0.800 (5)
C11L	0.8786 (3)	0.0161 (5)	0.06959 (18)	0.0745 (15)	0.800 (5)
H11D	0.9246	0.0650	0.0832	0.112*	0.800 (5)
H11E	0.8739	−0.0139	0.0316	0.112*	0.800 (5)
H11F	0.8817	−0.0517	0.0953	0.112*	0.800 (5)
C41	0.7366 (5)	0.1612 (8)	0.0655 (5)	0.055 (4)*	0.200 (5)
C42	0.7972 (6)	0.2407 (7)	0.0768 (5)	0.038 (4)*	0.200 (5)
H42	0.7889	0.3239	0.0803	0.046*	0.200 (5)
C43	0.8701 (5)	0.1984 (9)	0.0830 (5)	0.051 (4)*	0.200 (5)
H43	0.9115	0.2528	0.0907	0.061*	0.200 (5)
C44	0.8822 (5)	0.0767 (9)	0.0778 (5)	0.062 (5)*	0.200 (5)
H44	0.9320	0.0479	0.0820	0.075*	0.200 (5)
C45	0.8216 (6)	−0.0028 (7)	0.0665 (6)	0.060 (4)*	0.200 (5)
H45	0.8299	−0.0860	0.0629	0.072*	0.200 (5)
C46	0.7487 (5)	0.0394 (8)	0.0603 (6)	0.049 (6)*	0.200 (5)
H46	0.7073	−0.0149	0.0526	0.059*	0.200 (5)
C47	0.6630 (18)	0.205 (2)	0.0605 (13)	0.098 (10)*	0.200 (5)

### Atomic displacement parameters (Å^2^)

**Table:**

	*U*^11^	*U*^22^	*U*^33^	*U*^12^	*U*^13^	*U*^23^
Sm1	0.01623 (4)	0.01574 (4)	0.01597 (4)	−0.00062 (3)	0.00758 (3)	−0.00064 (3)
Si1	0.0259 (3)	0.0278 (3)	0.0180 (2)	0.0036 (2)	0.0057 (2)	0.00060 (19)
Si2	0.0272 (3)	0.0298 (3)	0.0179 (2)	0.0023 (2)	0.0087 (2)	−0.00247 (19)
N1	0.0165 (6)	0.0232 (7)	0.0158 (6)	−0.0016 (6)	0.0039 (5)	−0.0016 (6)
N2	0.0187 (7)	0.0236 (8)	0.0152 (7)	−0.0011 (6)	0.0062 (5)	−0.0009 (6)
O1L	0.0267 (7)	0.0185 (6)	0.0371 (8)	−0.0012 (5)	0.0135 (6)	−0.0028 (5)
C1L	0.0320 (11)	0.0212 (10)	0.0480 (13)	0.0030 (8)	0.0155 (10)	0.0002 (9)
C2L	0.0521 (15)	0.0253 (11)	0.088 (2)	0.0059 (11)	0.0357 (15)	0.0123 (13)
C3L	0.0451 (16)	0.0280 (14)	0.141 (4)	−0.0092 (11)	0.0318 (19)	−0.0171 (17)
C4L	0.0299 (11)	0.0251 (11)	0.0512 (14)	−0.0061 (8)	0.0132 (10)	−0.0071 (9)
C1	0.0174 (7)	0.0227 (9)	0.0187 (8)	−0.0003 (6)	0.0064 (6)	−0.0009 (6)
C2	0.0209 (8)	0.0200 (9)	0.0201 (8)	−0.0029 (6)	0.0084 (7)	−0.0041 (6)
C3	0.0214 (8)	0.0164 (8)	0.0242 (9)	−0.0014 (6)	0.0104 (7)	−0.0027 (6)
C4	0.0203 (8)	0.0185 (8)	0.0232 (9)	0.0006 (6)	0.0104 (7)	0.0036 (6)
C5	0.0217 (8)	0.0229 (9)	0.0198 (8)	0.0016 (7)	0.0095 (7)	0.0042 (7)
C6	0.0216 (8)	0.0226 (9)	0.0195 (8)	−0.0002 (7)	0.0106 (6)	−0.0002 (7)
C7	0.0191 (8)	0.0228 (8)	0.0208 (8)	0.0015 (7)	0.0101 (7)	−0.0016 (7)
C8	0.0167 (8)	0.0207 (8)	0.0207 (8)	0.0021 (6)	0.0065 (6)	0.0004 (7)
C9	0.0493 (14)	0.0501 (14)	0.0207 (10)	0.0122 (11)	0.0088 (9)	−0.0021 (9)
C10	0.0444 (13)	0.0346 (12)	0.0330 (12)	0.0013 (10)	0.0147 (10)	0.0097 (9)
C11	0.0265 (10)	0.0372 (12)	0.0339 (11)	0.0048 (8)	0.0010 (9)	−0.0007 (9)
C12	0.0668 (18)	0.0473 (15)	0.0345 (13)	−0.0208 (13)	0.0161 (13)	−0.0143 (11)
C13	0.0368 (12)	0.0508 (14)	0.0281 (11)	0.0104 (10)	0.0181 (9)	−0.0029 (9)
C14	0.0472 (14)	0.0646 (18)	0.0217 (10)	0.0222 (12)	0.0039 (10)	−0.0034 (10)
C15	0.0167 (7)	0.0200 (9)	0.0204 (8)	−0.0027 (6)	0.0081 (6)	−0.0030 (6)
C16	0.0168 (7)	0.0241 (10)	0.0196 (8)	0.0001 (6)	0.0044 (6)	−0.0030 (6)
C17	0.0213 (9)	0.0236 (9)	0.0212 (9)	−0.0001 (7)	0.0042 (7)	−0.0021 (7)
C18	0.0270 (10)	0.0343 (11)	0.0295 (11)	0.0007 (8)	−0.0009 (8)	0.0044 (8)
C19	0.0231 (10)	0.0487 (14)	0.0440 (13)	−0.0043 (10)	−0.0076 (9)	0.0074 (11)
C20	0.0209 (10)	0.0434 (13)	0.0448 (14)	−0.0090 (9)	0.0019 (9)	0.0052 (10)
C21	0.0214 (9)	0.0301 (11)	0.0262 (10)	−0.0030 (7)	0.0063 (7)	−0.0011 (8)
C22	0.0220 (9)	0.0252 (10)	0.0261 (10)	0.0023 (7)	0.0061 (7)	0.0048 (7)
C23	0.0340 (11)	0.0263 (11)	0.0421 (13)	−0.0010 (8)	0.0066 (10)	−0.0027 (9)
C24	0.0333 (11)	0.0452 (13)	0.0334 (12)	0.0061 (9)	0.0138 (10)	0.0135 (10)
C25	0.0232 (9)	0.0323 (11)	0.0268 (10)	−0.0077 (8)	0.0087 (8)	−0.0003 (8)
C26	0.0380 (12)	0.0345 (12)	0.0367 (13)	−0.0116 (9)	0.0062 (10)	0.0008 (9)
C27	0.0353 (12)	0.0526 (15)	0.0429 (14)	−0.0058 (10)	0.0230 (11)	0.0034 (11)
C28	0.0158 (7)	0.0324 (10)	0.0163 (7)	−0.0003 (7)	0.0079 (6)	−0.0013 (7)
C29	0.0162 (8)	0.0358 (11)	0.0201 (9)	0.0002 (7)	0.0083 (7)	−0.0057 (7)
C30	0.0289 (11)	0.0486 (14)	0.0243 (11)	0.0027 (9)	0.0105 (9)	−0.0123 (9)
C31	0.0362 (11)	0.0674 (16)	0.0164 (9)	0.0082 (11)	0.0099 (8)	−0.0056 (10)
C32	0.0333 (11)	0.0569 (15)	0.0180 (10)	0.0090 (10)	0.0117 (9)	0.0069 (9)
C33	0.0209 (9)	0.0391 (11)	0.0194 (9)	0.0012 (8)	0.0087 (7)	0.0028 (8)
C34	0.0259 (9)	0.0289 (10)	0.0276 (10)	−0.0001 (7)	0.0134 (8)	−0.0083 (8)
C35	0.0315 (11)	0.0360 (12)	0.0379 (12)	0.0075 (9)	0.0124 (9)	−0.0014 (9)
C36	0.0368 (12)	0.0381 (13)	0.0464 (14)	−0.0073 (9)	0.0196 (11)	−0.0139 (10)
C37	0.0393 (12)	0.0346 (11)	0.0273 (11)	0.0022 (9)	0.0184 (9)	0.0079 (8)
C38	0.0524 (15)	0.0481 (15)	0.0315 (12)	−0.0134 (12)	0.0125 (11)	0.0088 (10)
C39	0.0555 (16)	0.0442 (14)	0.0435 (14)	0.0099 (12)	0.0279 (12)	0.0176 (11)
C5L	0.072 (3)	0.060 (2)	0.0218 (15)	−0.0081 (18)	0.0130 (15)	0.0029 (13)
C6L	0.052 (2)	0.047 (2)	0.0368 (18)	−0.0155 (19)	0.0085 (15)	0.0015 (15)
C7L	0.067 (3)	0.057 (2)	0.055 (2)	−0.014 (2)	0.0138 (19)	0.0053 (18)
C8L	0.062 (3)	0.091 (4)	0.038 (2)	−0.005 (3)	0.0100 (19)	0.010 (2)
C9L	0.066 (3)	0.090 (3)	0.0342 (18)	−0.038 (2)	0.0181 (18)	−0.0124 (18)
C10L	0.115 (5)	0.073 (3)	0.0235 (18)	−0.056 (3)	0.026 (2)	−0.0122 (16)
C11L	0.100 (4)	0.086 (4)	0.035 (2)	0.017 (3)	0.016 (2)	−0.005 (2)

### Geometric parameters (Å, °)

**Table:**

Sm1—N2	2.4699 (15)	C20—C21	1.397 (3)
Sm1—O1L	2.4731 (14)	C20—H20A	0.9500
Sm1—N1	2.5555 (15)	C21—C25	1.523 (3)
Sm1—C2	2.6160 (19)	C22—C23	1.530 (3)
Sm1—C8	2.6297 (18)	C22—C24	1.531 (3)
Sm1—C7	2.6369 (18)	C22—H22A	1.0000
Sm1—C1	2.6377 (19)	C23—H23A	0.9800
Sm1—C5	2.6439 (18)	C23—H23B	0.9800
Sm1—C3	2.6504 (18)	C23—H23C	0.9800
Sm1—C4	2.6549 (18)	C24—H24A	0.9800
Sm1—C6	2.6668 (17)	C24—H24B	0.9800
Sm1—C15	2.8796 (17)	C24—H24C	0.9800
Si1—C10	1.867 (2)	C25—C26	1.533 (3)
Si1—C9	1.868 (2)	C25—C27	1.536 (3)
Si1—C11	1.873 (2)	C25—H25A	1.0000
Si1—C1	1.8844 (19)	C26—H26A	0.9800
Si2—C13	1.867 (2)	C26—H26B	0.9800
Si2—C14	1.868 (2)	C26—H26C	0.9800
Si2—C12	1.871 (3)	C27—H27A	0.9800
Si2—C6	1.8784 (19)	C27—H27B	0.9800
N1—C15	1.323 (2)	C27—H27C	0.9800
N1—C16	1.428 (2)	C28—C29	1.406 (3)
N2—C15	1.326 (2)	C28—C33	1.413 (3)
N2—C28	1.424 (2)	C29—C30	1.397 (3)
O1L—C4L	1.451 (2)	C29—C34	1.524 (3)
O1L—C1L	1.466 (2)	C30—C31	1.376 (4)
C1L—C2L	1.490 (3)	C30—H30A	0.9500
C1L—H1LA	0.9900	C31—C32	1.383 (4)
C1L—H1LB	0.9900	C31—H31A	0.9500
C2L—C3L	1.479 (4)	C32—C33	1.389 (3)
C2L—H2LA	0.9900	C32—H32A	0.9500
C2L—H2LB	0.9900	C33—C37	1.520 (3)
C3L—C4L	1.500 (4)	C34—C36	1.525 (3)
C3L—H3LA	0.9900	C34—C35	1.532 (3)
C3L—H3LB	0.9900	C34—H34A	1.0000
C4L—H4LA	0.9900	C35—H35A	0.9800
C4L—H4LB	0.9900	C35—H35B	0.9800
C1—C2	1.421 (3)	C35—H35C	0.9800
C1—C8	1.425 (2)	C36—H36A	0.9800
C2—C3	1.410 (3)	C36—H36B	0.9800
C2—H2	0.9500	C36—H36C	0.9800
C3—C4	1.410 (3)	C37—C39	1.534 (3)
C3—H3	0.9500	C37—C38	1.536 (3)
C4—C5	1.410 (3)	C37—H37A	1.0000
C4—H4	0.9500	C38—H38A	0.9800
C5—C6	1.422 (3)	C38—H38B	0.9800
C5—H5	0.9500	C38—H38C	0.9800
C6—C7	1.428 (3)	C39—H39A	0.9800
C7—C8	1.414 (3)	C39—H39B	0.9800
C7—H7	0.9500	C39—H39C	0.9800
C8—H8	0.9500	C5L—C6L	1.3900
C9—H9A	0.9800	C5L—C10L	1.3900
C9—H9B	0.9800	C5L—C11L	1.497 (5)
C9—H9C	0.9800	C6L—C7L	1.3900
C10—H10A	0.9800	C6L—H6L	0.9500
C10—H10B	0.9800	C7L—C8L	1.3900
C10—H10C	0.9800	C7L—H7L	0.9500
C11—H11A	0.9800	C8L—C9L	1.3900
C11—H11B	0.9800	C8L—H8L	0.9500
C11—H11C	0.9800	C9L—C10L	1.3900
C12—H12A	0.9800	C9L—H9L	0.9500
C12—H12B	0.9800	C10L—H10L	0.9500
C12—H12C	0.9800	C11L—H11D	0.9800
C13—H13A	0.9800	C11L—H11E	0.9800
C13—H13B	0.9800	C11L—H11F	0.9800
C13—H13C	0.9800	C41—C42	1.3900
C14—H14A	0.9800	C41—C46	1.3900
C14—H14B	0.9800	C41—C47	1.41 (3)
C14—H14C	0.9800	C42—C43	1.3900
C15—H15	0.9500	C42—H42	0.9500
C16—C21	1.416 (3)	C43—C44	1.3900
C16—C17	1.419 (3)	C43—H43	0.9500
C17—C18	1.389 (3)	C44—C45	1.3900
C17—C22	1.511 (3)	C44—H44	0.9500
C18—C19	1.381 (3)	C45—C46	1.3900
C18—H18A	0.9500	C45—H45	0.9500
C19—C20	1.378 (3)	C46—H46	0.9500
C19—H19A	0.9500		
			
N2—Sm1—O1L	86.99 (5)	H11A—C11—H11B	109.5
N2—Sm1—N1	54.31 (5)	Si1—C11—H11C	109.5
O1L—Sm1—N1	85.08 (5)	H11A—C11—H11C	109.5
N2—Sm1—C2	90.50 (5)	H11B—C11—H11C	109.5
O1L—Sm1—C2	133.79 (5)	Si2—C12—H12A	109.5
N1—Sm1—C2	128.89 (5)	Si2—C12—H12B	109.5
N2—Sm1—C8	124.06 (6)	H12A—C12—H12B	109.5
O1L—Sm1—C8	84.70 (5)	Si2—C12—H12C	109.5
N1—Sm1—C8	169.74 (6)	H12A—C12—H12C	109.5
C2—Sm1—C8	59.23 (6)	H12B—C12—H12C	109.5
N2—Sm1—C7	153.23 (6)	Si2—C13—H13A	109.5
O1L—Sm1—C7	81.34 (5)	Si2—C13—H13B	109.5
N1—Sm1—C7	146.91 (5)	H13A—C13—H13B	109.5
C2—Sm1—C7	80.63 (6)	Si2—C13—H13C	109.5
C8—Sm1—C7	31.16 (6)	H13A—C13—H13C	109.5
N2—Sm1—C1	100.20 (5)	H13B—C13—H13C	109.5
O1L—Sm1—C1	104.13 (5)	Si2—C14—H14A	109.5
N1—Sm1—C1	152.96 (5)	Si2—C14—H14B	109.5
C2—Sm1—C1	31.38 (6)	H14A—C14—H14B	109.5
C8—Sm1—C1	31.39 (5)	Si2—C14—H14C	109.5
C7—Sm1—C1	60.11 (6)	H14A—C14—H14C	109.5
N2—Sm1—C5	145.04 (5)	H14B—C14—H14C	109.5
O1L—Sm1—C5	122.77 (5)	N1—C15—N2	120.10 (15)
N1—Sm1—C5	106.09 (6)	N1—C15—Sm1	62.55 (9)
C2—Sm1—C5	81.41 (6)	N2—C15—Sm1	58.86 (9)
C8—Sm1—C5	80.38 (6)	N1—C15—H15	120.0
C7—Sm1—C5	58.74 (6)	N2—C15—H15	120.0
C1—Sm1—C5	90.40 (6)	Sm1—C15—H15	168.4
N2—Sm1—C3	97.91 (5)	C21—C16—C17	119.70 (16)
O1L—Sm1—C3	163.42 (5)	C21—C16—N1	122.01 (16)
N1—Sm1—C3	110.66 (5)	C17—C16—N1	118.29 (16)
C2—Sm1—C3	31.05 (6)	C18—C17—C16	119.07 (18)
C8—Sm1—C3	79.43 (6)	C18—C17—C22	120.12 (18)
C7—Sm1—C3	87.45 (6)	C16—C17—C22	120.65 (16)
C1—Sm1—C3	59.48 (6)	C19—C18—C17	121.5 (2)
C5—Sm1—C3	59.24 (6)	C19—C18—H18A	119.2
N2—Sm1—C4	118.02 (5)	C17—C18—H18A	119.2
O1L—Sm1—C4	153.61 (5)	C20—C19—C18	119.3 (2)
N1—Sm1—C4	102.26 (5)	C20—C19—H19A	120.3
C2—Sm1—C4	59.30 (6)	C18—C19—H19A	120.3
C8—Sm1—C4	87.38 (6)	C19—C20—C21	122.0 (2)
C7—Sm1—C4	78.92 (6)	C19—C20—H20A	119.0
C1—Sm1—C4	80.86 (6)	C21—C20—H20A	119.0
C5—Sm1—C4	30.86 (6)	C20—C21—C16	118.33 (19)
C3—Sm1—C4	30.82 (6)	C20—C21—C25	117.27 (18)
N2—Sm1—C6	175.52 (5)	C16—C21—C25	124.40 (17)
O1L—Sm1—C6	95.43 (5)	C17—C22—C23	109.41 (17)
N1—Sm1—C6	122.05 (5)	C17—C22—C24	114.06 (18)
C2—Sm1—C6	90.53 (6)	C23—C22—C24	110.47 (18)
C8—Sm1—C6	60.04 (6)	C17—C22—H22A	107.6
C7—Sm1—C6	31.22 (6)	C23—C22—H22A	107.6
C1—Sm1—C6	82.89 (6)	C24—C22—H22A	107.6
C5—Sm1—C6	31.06 (6)	C22—C23—H23A	109.5
C3—Sm1—C6	80.81 (6)	C22—C23—H23B	109.5
C4—Sm1—C6	59.06 (6)	H23A—C23—H23B	109.5
N2—Sm1—C15	27.35 (5)	C22—C23—H23C	109.5
O1L—Sm1—C15	88.92 (5)	H23A—C23—H23C	109.5
N1—Sm1—C15	27.34 (5)	H23B—C23—H23C	109.5
C2—Sm1—C15	108.63 (5)	C22—C24—H24A	109.5
C8—Sm1—C15	151.24 (5)	C22—C24—H24B	109.5
C7—Sm1—C15	169.76 (6)	H24A—C24—H24B	109.5
C1—Sm1—C15	126.09 (5)	C22—C24—H24C	109.5
C5—Sm1—C15	125.79 (6)	H24A—C24—H24C	109.5
C3—Sm1—C15	102.73 (5)	H24B—C24—H24C	109.5
C4—Sm1—C15	109.33 (5)	C21—C25—C26	112.25 (18)
C6—Sm1—C15	148.67 (5)	C21—C25—C27	111.45 (19)
C10—Si1—C9	108.28 (12)	C26—C25—C27	110.28 (18)
C10—Si1—C11	106.84 (11)	C21—C25—H25A	107.5
C9—Si1—C11	110.13 (11)	C26—C25—H25A	107.5
C10—Si1—C1	110.74 (10)	C27—C25—H25A	107.5
C9—Si1—C1	111.09 (10)	C25—C26—H26A	109.5
C11—Si1—C1	109.66 (10)	C25—C26—H26B	109.5
C13—Si2—C14	109.18 (12)	H26A—C26—H26B	109.5
C13—Si2—C12	108.30 (13)	C25—C26—H26C	109.5
C14—Si2—C12	108.59 (14)	H26A—C26—H26C	109.5
C13—Si2—C6	108.46 (10)	H26B—C26—H26C	109.5
C14—Si2—C6	112.37 (10)	C25—C27—H27A	109.5
C12—Si2—C6	109.86 (10)	C25—C27—H27B	109.5
C15—N1—C16	117.02 (14)	H27A—C27—H27B	109.5
C15—N1—Sm1	90.10 (10)	C25—C27—H27C	109.5
C16—N1—Sm1	152.46 (11)	H27A—C27—H27C	109.5
C15—N2—C28	117.72 (14)	H27B—C27—H27C	109.5
C15—N2—Sm1	93.79 (10)	C29—C28—C33	120.34 (17)
C28—N2—Sm1	142.32 (11)	C29—C28—N2	121.02 (17)
C4L—O1L—C1L	108.72 (15)	C33—C28—N2	118.63 (17)
C4L—O1L—Sm1	120.88 (12)	C30—C29—C28	118.33 (19)
C1L—O1L—Sm1	129.95 (12)	C30—C29—C34	118.85 (19)
O1L—C1L—C2L	104.99 (18)	C28—C29—C34	122.81 (16)
O1L—C1L—H1LA	110.7	C31—C30—C29	121.7 (2)
C2L—C1L—H1LA	110.7	C31—C30—H30A	119.2
O1L—C1L—H1LB	110.7	C29—C30—H30A	119.2
C2L—C1L—H1LB	110.7	C30—C31—C32	119.56 (19)
H1LA—C1L—H1LB	108.8	C30—C31—H31A	120.2
C3L—C2L—C1L	103.6 (2)	C32—C31—H31A	120.2
C3L—C2L—H2LA	111.0	C31—C32—C33	121.3 (2)
C1L—C2L—H2LA	111.0	C31—C32—H32A	119.3
C3L—C2L—H2LB	111.0	C33—C32—H32A	119.3
C1L—C2L—H2LB	111.0	C32—C33—C28	118.7 (2)
H2LA—C2L—H2LB	109.0	C32—C33—C37	119.99 (19)
C2L—C3L—C4L	104.1 (2)	C28—C33—C37	121.19 (17)
C2L—C3L—H3LA	110.9	C29—C34—C36	112.09 (19)
C4L—C3L—H3LA	110.9	C29—C34—C35	111.93 (17)
C2L—C3L—H3LB	110.9	C36—C34—C35	109.20 (18)
C4L—C3L—H3LB	110.9	C29—C34—H34A	107.8
H3LA—C3L—H3LB	109.0	C36—C34—H34A	107.8
O1L—C4L—C3L	105.69 (19)	C35—C34—H34A	107.8
O1L—C4L—H4LA	110.6	C34—C35—H35A	109.5
C3L—C4L—H4LA	110.6	C34—C35—H35B	109.5
O1L—C4L—H4LB	110.6	H35A—C35—H35B	109.5
C3L—C4L—H4LB	110.6	C34—C35—H35C	109.5
H4LA—C4L—H4LB	108.7	H35A—C35—H35C	109.5
C2—C1—C8	131.28 (17)	H35B—C35—H35C	109.5
C2—C1—Si1	116.16 (13)	C34—C36—H36A	109.5
C8—C1—Si1	112.56 (13)	C34—C36—H36B	109.5
C2—C1—Sm1	73.47 (10)	H36A—C36—H36B	109.5
C8—C1—Sm1	73.99 (10)	C34—C36—H36C	109.5
Si1—C1—Sm1	132.91 (9)	H36A—C36—H36C	109.5
C3—C2—C1	135.86 (17)	H36B—C36—H36C	109.5
C3—C2—Sm1	75.82 (11)	C33—C37—C39	113.9 (2)
C1—C2—Sm1	75.16 (10)	C33—C37—C38	110.19 (19)
C3—C2—H2	112.1	C39—C37—C38	109.1 (2)
C1—C2—H2	112.1	C33—C37—H37A	107.8
Sm1—C2—H2	131.8	C39—C37—H37A	107.8
C4—C3—C2	135.30 (17)	C38—C37—H37A	107.8
C4—C3—Sm1	74.76 (10)	C37—C38—H38A	109.5
C2—C3—Sm1	73.13 (10)	C37—C38—H38B	109.5
C4—C3—H3	112.3	H38A—C38—H38B	109.5
C2—C3—H3	112.3	C37—C38—H38C	109.5
Sm1—C3—H3	136.7	H38A—C38—H38C	109.5
C3—C4—C5	136.25 (17)	H38B—C38—H38C	109.5
C3—C4—Sm1	74.42 (10)	C37—C39—H39A	109.5
C5—C4—Sm1	74.14 (10)	C37—C39—H39B	109.5
C3—C4—H4	111.9	H39A—C39—H39B	109.5
C5—C4—H4	111.9	C37—C39—H39C	109.5
Sm1—C4—H4	136.7	H39A—C39—H39C	109.5
C4—C5—C6	135.70 (18)	H39B—C39—H39C	109.5
C4—C5—Sm1	75.00 (10)	C6L—C5L—C10L	120.0
C6—C5—Sm1	75.36 (10)	C6L—C5L—C11L	120.9 (3)
C4—C5—H5	112.2	C10L—C5L—C11L	119.1 (3)
C6—C5—H5	112.2	C7L—C6L—C5L	120.0
Sm1—C5—H5	132.7	C7L—C6L—H6L	120.0
C5—C6—C7	130.70 (17)	C5L—C6L—H6L	120.0
C5—C6—Si2	116.62 (14)	C6L—C7L—C8L	120.0
C7—C6—Si2	112.67 (13)	C6L—C7L—H7L	120.0
C5—C6—Sm1	73.58 (10)	C8L—C7L—H7L	120.0
C7—C6—Sm1	73.23 (10)	C9L—C8L—C7L	120.0
Si2—C6—Sm1	133.29 (9)	C9L—C8L—H8L	120.0
C8—C7—C6	137.64 (17)	C7L—C8L—H8L	120.0
C8—C7—Sm1	74.14 (10)	C8L—C9L—C10L	120.0
C6—C7—Sm1	75.55 (10)	C8L—C9L—H9L	120.0
C8—C7—H7	111.2	C10L—C9L—H9L	120.0
C6—C7—H7	111.2	C9L—C10L—C5L	120.0
Sm1—C7—H7	136.4	C9L—C10L—H10L	120.0
C7—C8—C1	136.98 (17)	C5L—C10L—H10L	120.0
C7—C8—Sm1	74.70 (10)	C42—C41—C46	120.0
C1—C8—Sm1	74.61 (10)	C42—C41—C47	119.6 (7)
C7—C8—H8	111.5	C46—C41—C47	120.4 (7)
C1—C8—H8	111.5	C41—C42—C43	120.0
Sm1—C8—H8	136.2	C41—C42—H42	120.0
Si1—C9—H9A	109.5	C43—C42—H42	120.0
Si1—C9—H9B	109.5	C44—C43—C42	120.0
H9A—C9—H9B	109.5	C44—C43—H43	120.0
Si1—C9—H9C	109.5	C42—C43—H43	120.0
H9A—C9—H9C	109.5	C45—C44—C43	120.0
H9B—C9—H9C	109.5	C45—C44—H44	120.0
Si1—C10—H10A	109.5	C43—C44—H44	120.0
Si1—C10—H10B	109.5	C44—C45—C46	120.0
H10A—C10—H10B	109.5	C44—C45—H45	120.0
Si1—C10—H10C	109.5	C46—C45—H45	120.0
H10A—C10—H10C	109.5	C45—C46—C41	120.0
H10B—C10—H10C	109.5	C45—C46—H46	120.0
Si1—C11—H11A	109.5	C41—C46—H46	120.0
Si1—C11—H11B	109.5		
			
N2—Sm1—N1—C15	−7.36 (10)	C2—Sm1—C5—C6	−105.61 (11)
O1L—Sm1—N1—C15	−97.18 (11)	C8—Sm1—C5—C6	−45.55 (11)
C2—Sm1—N1—C15	48.20 (13)	C7—Sm1—C5—C6	−21.40 (10)
C8—Sm1—N1—C15	−91.8 (3)	C1—Sm1—C5—C6	−75.44 (11)
C7—Sm1—N1—C15	−163.04 (11)	C3—Sm1—C5—C6	−128.91 (12)
C1—Sm1—N1—C15	14.56 (18)	C4—Sm1—C5—C6	−146.70 (17)
C5—Sm1—N1—C15	140.10 (11)	C15—Sm1—C5—C6	147.57 (10)
C3—Sm1—N1—C15	77.50 (11)	C4—C5—C6—C7	0.0 (4)
C4—Sm1—N1—C15	108.48 (11)	Sm1—C5—C6—C7	49.45 (19)
C6—Sm1—N1—C15	169.48 (10)	C4—C5—C6—Si2	179.90 (18)
N2—Sm1—N1—C16	−177.8 (3)	Sm1—C5—C6—Si2	−130.69 (11)
O1L—Sm1—N1—C16	92.4 (3)	C4—C5—C6—Sm1	−49.4 (2)
C2—Sm1—N1—C16	−122.2 (2)	C13—Si2—C6—C5	−117.72 (15)
C8—Sm1—N1—C16	97.7 (4)	C14—Si2—C6—C5	3.08 (19)
C7—Sm1—N1—C16	26.5 (3)	C12—Si2—C6—C5	124.09 (17)
C1—Sm1—N1—C16	−155.9 (2)	C13—Si2—C6—C7	62.17 (16)
C5—Sm1—N1—C16	−30.4 (3)	C14—Si2—C6—C7	−177.03 (15)
C3—Sm1—N1—C16	−93.0 (3)	C12—Si2—C6—C7	−56.02 (17)
C4—Sm1—N1—C16	−62.0 (3)	C13—Si2—C6—Sm1	150.11 (13)
C6—Sm1—N1—C16	−1.0 (3)	C14—Si2—C6—Sm1	−89.10 (16)
C15—Sm1—N1—C16	−170.4 (3)	C12—Si2—C6—Sm1	31.91 (17)
O1L—Sm1—N2—C15	93.46 (11)	O1L—Sm1—C6—C5	−153.64 (11)
N1—Sm1—N2—C15	7.35 (10)	N1—Sm1—C6—C5	−66.06 (12)
C2—Sm1—N2—C15	−132.71 (11)	C2—Sm1—C6—C5	72.24 (11)
C8—Sm1—N2—C15	174.99 (10)	C8—Sm1—C6—C5	125.67 (12)
C7—Sm1—N2—C15	157.41 (12)	C7—Sm1—C6—C5	143.02 (17)
C1—Sm1—N2—C15	−162.72 (11)	C1—Sm1—C6—C5	102.75 (11)
C5—Sm1—N2—C15	−57.08 (15)	C3—Sm1—C6—C5	42.63 (11)
C3—Sm1—N2—C15	−102.45 (11)	C4—Sm1—C6—C5	19.17 (10)
C4—Sm1—N2—C15	−77.71 (12)	C15—Sm1—C6—C5	−56.78 (15)
O1L—Sm1—N2—C28	−118.5 (2)	O1L—Sm1—C6—C7	63.34 (11)
N1—Sm1—N2—C28	155.4 (2)	N1—Sm1—C6—C7	150.93 (10)
C2—Sm1—N2—C28	15.3 (2)	C2—Sm1—C6—C7	−70.77 (11)
C8—Sm1—N2—C28	−37.0 (2)	C8—Sm1—C6—C7	−17.34 (10)
C7—Sm1—N2—C28	−54.6 (3)	C1—Sm1—C6—C7	−40.27 (11)
C1—Sm1—N2—C28	−14.7 (2)	C5—Sm1—C6—C7	−143.02 (17)
C5—Sm1—N2—C28	90.9 (2)	C3—Sm1—C6—C7	−100.38 (11)
C3—Sm1—N2—C28	45.6 (2)	C4—Sm1—C6—C7	−123.84 (12)
C4—Sm1—N2—C28	70.3 (2)	C15—Sm1—C6—C7	160.21 (11)
C15—Sm1—N2—C28	148.0 (3)	O1L—Sm1—C6—Si2	−42.28 (12)
N2—Sm1—O1L—C4L	−51.03 (16)	N1—Sm1—C6—Si2	45.31 (14)
N1—Sm1—O1L—C4L	3.40 (15)	C2—Sm1—C6—Si2	−176.39 (12)
C2—Sm1—O1L—C4L	−138.83 (15)	C8—Sm1—C6—Si2	−122.96 (14)
C8—Sm1—O1L—C4L	−175.65 (16)	C7—Sm1—C6—Si2	−105.62 (17)
C7—Sm1—O1L—C4L	153.14 (16)	C1—Sm1—C6—Si2	−145.89 (13)
C1—Sm1—O1L—C4L	−150.79 (15)	C5—Sm1—C6—Si2	111.36 (17)
C5—Sm1—O1L—C4L	109.39 (16)	C3—Sm1—C6—Si2	154.00 (13)
C3—Sm1—O1L—C4L	−158.88 (19)	C4—Sm1—C6—Si2	130.54 (14)
C4—Sm1—O1L—C4L	111.23 (17)	C15—Sm1—C6—Si2	54.59 (18)
C6—Sm1—O1L—C4L	125.19 (16)	C5—C6—C7—C8	−4.1 (4)
C15—Sm1—O1L—C4L	−23.72 (16)	Si2—C6—C7—C8	176.01 (19)
N2—Sm1—O1L—C1L	120.43 (17)	Sm1—C6—C7—C8	45.4 (2)
N1—Sm1—O1L—C1L	174.85 (17)	C5—C6—C7—Sm1	−49.57 (19)
C2—Sm1—O1L—C1L	32.62 (19)	Si2—C6—C7—Sm1	130.57 (10)
C8—Sm1—O1L—C1L	−4.19 (17)	N2—Sm1—C7—C8	28.92 (19)
C7—Sm1—O1L—C1L	−35.41 (17)	O1L—Sm1—C7—C8	94.09 (11)
C1—Sm1—O1L—C1L	20.66 (17)	N1—Sm1—C7—C8	160.97 (11)
C5—Sm1—O1L—C1L	−79.16 (18)	C2—Sm1—C7—C8	−43.18 (11)
C3—Sm1—O1L—C1L	12.6 (3)	C1—Sm1—C7—C8	−17.77 (10)
C4—Sm1—O1L—C1L	−77.3 (2)	C5—Sm1—C7—C8	−128.77 (13)
C6—Sm1—O1L—C1L	−63.35 (17)	C3—Sm1—C7—C8	−73.66 (11)
C15—Sm1—O1L—C1L	147.73 (17)	C4—Sm1—C7—C8	−103.52 (12)
C4L—O1L—C1L—C2L	15.6 (3)	C6—Sm1—C7—C8	−150.06 (17)
Sm1—O1L—C1L—C2L	−156.70 (17)	C15—Sm1—C7—C8	112.1 (3)
O1L—C1L—C2L—C3L	−31.8 (3)	N2—Sm1—C7—C6	178.98 (11)
C1L—C2L—C3L—C4L	35.9 (3)	O1L—Sm1—C7—C6	−115.85 (11)
C1L—O1L—C4L—C3L	6.7 (3)	N1—Sm1—C7—C6	−48.97 (16)
Sm1—O1L—C4L—C3L	179.8 (2)	C2—Sm1—C7—C6	106.88 (11)
C2L—C3L—C4L—O1L	−26.6 (3)	C8—Sm1—C7—C6	150.06 (17)
C10—Si1—C1—C2	−128.77 (15)	C1—Sm1—C7—C6	132.29 (13)
C9—Si1—C1—C2	−8.41 (17)	C5—Sm1—C7—C6	21.29 (10)
C11—Si1—C1—C2	113.56 (15)	C3—Sm1—C7—C6	76.40 (11)
C10—Si1—C1—C8	51.36 (16)	C4—Sm1—C7—C6	46.54 (11)
C9—Si1—C1—C8	171.73 (14)	C15—Sm1—C7—C6	−97.9 (3)
C11—Si1—C1—C8	−66.30 (15)	C6—C7—C8—C1	0.0 (4)
C10—Si1—C1—Sm1	−37.41 (15)	Sm1—C7—C8—C1	45.9 (2)
C9—Si1—C1—Sm1	82.95 (15)	C6—C7—C8—Sm1	−45.8 (2)
C11—Si1—C1—Sm1	−155.07 (12)	C2—C1—C8—C7	3.9 (4)
N2—Sm1—C1—C2	73.86 (11)	Si1—C1—C8—C7	−176.27 (19)
O1L—Sm1—C1—C2	163.31 (10)	Sm1—C1—C8—C7	−45.9 (2)
N1—Sm1—C1—C2	55.93 (16)	C2—C1—C8—Sm1	49.78 (18)
C8—Sm1—C1—C2	−143.23 (16)	Si1—C1—C8—Sm1	−130.38 (10)
C7—Sm1—C1—C2	−125.59 (12)	N2—Sm1—C8—C7	−164.76 (10)
C5—Sm1—C1—C2	−72.64 (11)	O1L—Sm1—C8—C7	−82.02 (11)
C3—Sm1—C1—C2	−19.37 (10)	N1—Sm1—C8—C7	−87.4 (3)
C4—Sm1—C1—C2	−43.17 (10)	C2—Sm1—C8—C7	128.21 (13)
C6—Sm1—C1—C2	−102.85 (11)	C1—Sm1—C8—C7	149.47 (17)
C15—Sm1—C1—C2	64.14 (12)	C5—Sm1—C8—C7	42.53 (11)
N2—Sm1—C1—C8	−142.91 (10)	C3—Sm1—C8—C7	102.78 (12)
O1L—Sm1—C1—C8	−53.46 (11)	C4—Sm1—C8—C7	72.78 (11)
N1—Sm1—C1—C8	−160.84 (12)	C6—Sm1—C8—C7	17.37 (10)
C2—Sm1—C1—C8	143.23 (16)	C15—Sm1—C8—C7	−159.98 (11)
C7—Sm1—C1—C8	17.64 (10)	N2—Sm1—C8—C1	45.77 (12)
C5—Sm1—C1—C8	70.59 (11)	O1L—Sm1—C8—C1	128.51 (11)
C3—Sm1—C1—C8	123.87 (12)	N1—Sm1—C8—C1	123.2 (3)
C4—Sm1—C1—C8	100.06 (11)	C2—Sm1—C8—C1	−21.27 (10)
C6—Sm1—C1—C8	40.38 (11)	C7—Sm1—C8—C1	−149.47 (17)
C15—Sm1—C1—C8	−152.63 (10)	C5—Sm1—C8—C1	−106.95 (11)
N2—Sm1—C1—Si1	−36.74 (12)	C3—Sm1—C8—C1	−46.69 (10)
O1L—Sm1—C1—Si1	52.70 (12)	C4—Sm1—C8—C1	−76.69 (11)
N1—Sm1—C1—Si1	−54.68 (19)	C6—Sm1—C8—C1	−132.10 (12)
C2—Sm1—C1—Si1	−110.61 (16)	C15—Sm1—C8—C1	50.55 (16)
C8—Sm1—C1—Si1	106.16 (17)	C16—N1—C15—N2	−172.00 (16)
C7—Sm1—C1—Si1	123.81 (14)	Sm1—N1—C15—N2	12.94 (17)
C5—Sm1—C1—Si1	176.75 (12)	C16—N1—C15—Sm1	175.06 (17)
C3—Sm1—C1—Si1	−129.97 (13)	C28—N2—C15—N1	−171.97 (16)
C4—Sm1—C1—Si1	−153.78 (12)	Sm1—N2—C15—N1	−13.42 (18)
C6—Sm1—C1—Si1	146.54 (12)	C28—N2—C15—Sm1	−158.55 (18)
C15—Sm1—C1—Si1	−46.47 (14)	N2—Sm1—C15—N1	166.92 (17)
C8—C1—C2—C3	0.5 (3)	O1L—Sm1—C15—N1	81.37 (11)
Si1—C1—C2—C3	−179.36 (17)	C2—Sm1—C15—N1	−142.24 (11)
Sm1—C1—C2—C3	50.4 (2)	C8—Sm1—C15—N1	158.28 (12)
C8—C1—C2—Sm1	−49.96 (18)	C7—Sm1—C15—N1	63.6 (3)
Si1—C1—C2—Sm1	130.20 (11)	C1—Sm1—C15—N1	−171.87 (10)
N2—Sm1—C2—C3	104.62 (10)	C5—Sm1—C15—N1	−49.45 (13)
O1L—Sm1—C2—C3	−169.07 (9)	C3—Sm1—C15—N1	−110.53 (11)
N1—Sm1—C2—C3	62.56 (12)	C4—Sm1—C15—N1	−79.15 (11)
C8—Sm1—C2—C3	−125.09 (12)	C6—Sm1—C15—N1	−17.31 (16)
C7—Sm1—C2—C3	−100.76 (11)	O1L—Sm1—C15—N2	−85.55 (11)
C1—Sm1—C2—C3	−146.37 (16)	N1—Sm1—C15—N2	−166.92 (17)
C5—Sm1—C2—C3	−41.23 (10)	C2—Sm1—C15—N2	50.84 (12)
C4—Sm1—C2—C3	−18.14 (10)	C8—Sm1—C15—N2	−8.64 (17)
C6—Sm1—C2—C3	−71.02 (11)	C7—Sm1—C15—N2	−103.3 (3)
C15—Sm1—C2—C3	83.75 (11)	C1—Sm1—C15—N2	21.21 (13)
N2—Sm1—C2—C1	−109.01 (10)	C5—Sm1—C15—N2	143.63 (11)
O1L—Sm1—C2—C1	−22.70 (13)	C3—Sm1—C15—N2	82.55 (11)
N1—Sm1—C2—C1	−151.07 (9)	C4—Sm1—C15—N2	113.93 (11)
C8—Sm1—C2—C1	21.28 (9)	C6—Sm1—C15—N2	175.77 (11)
C7—Sm1—C2—C1	45.61 (10)	C15—N1—C16—C21	52.3 (2)
C5—Sm1—C2—C1	105.14 (11)	Sm1—N1—C16—C21	−138.5 (2)
C3—Sm1—C2—C1	146.37 (16)	C15—N1—C16—C17	−128.79 (18)
C4—Sm1—C2—C1	128.23 (12)	Sm1—N1—C16—C17	40.5 (3)
C6—Sm1—C2—C1	75.35 (10)	C21—C16—C17—C18	−0.8 (3)
C15—Sm1—C2—C1	−129.88 (10)	N1—C16—C17—C18	−179.80 (18)
C1—C2—C3—C4	−4.4 (4)	C21—C16—C17—C22	−176.26 (17)
Sm1—C2—C3—C4	45.8 (2)	N1—C16—C17—C22	4.8 (3)
C1—C2—C3—Sm1	−50.2 (2)	C16—C17—C18—C19	−0.8 (3)
N2—Sm1—C3—C4	133.86 (10)	C22—C17—C18—C19	174.6 (2)
O1L—Sm1—C3—C4	−119.81 (18)	C17—C18—C19—C20	1.4 (4)
N1—Sm1—C3—C4	79.10 (11)	C18—C19—C20—C21	−0.3 (4)
C2—Sm1—C3—C4	−148.49 (16)	C19—C20—C21—C16	−1.3 (4)
C8—Sm1—C3—C4	−102.83 (11)	C19—C20—C21—C25	177.9 (2)
C7—Sm1—C3—C4	−72.49 (11)	C17—C16—C21—C20	1.8 (3)
C1—Sm1—C3—C4	−128.93 (12)	N1—C16—C21—C20	−179.24 (19)
C5—Sm1—C3—C4	−17.81 (10)	C17—C16—C21—C25	−177.29 (18)
C6—Sm1—C3—C4	−41.80 (10)	N1—C16—C21—C25	1.6 (3)
C15—Sm1—C3—C4	106.47 (11)	C18—C17—C22—C23	−91.7 (2)
N2—Sm1—C3—C2	−77.66 (11)	C16—C17—C22—C23	83.7 (2)
O1L—Sm1—C3—C2	28.7 (2)	C18—C17—C22—C24	32.6 (3)
N1—Sm1—C3—C2	−132.42 (10)	C16—C17—C22—C24	−152.04 (18)
C8—Sm1—C3—C2	45.66 (10)	C20—C21—C25—C26	−59.2 (3)
C7—Sm1—C3—C2	76.00 (11)	C16—C21—C25—C26	120.0 (2)
C1—Sm1—C3—C2	19.55 (10)	C20—C21—C25—C27	65.1 (3)
C5—Sm1—C3—C2	130.68 (12)	C16—C21—C25—C27	−115.8 (2)
C4—Sm1—C3—C2	148.49 (16)	C15—N2—C28—C29	66.6 (2)
C6—Sm1—C3—C2	106.68 (11)	Sm1—N2—C28—C29	−76.8 (2)
C15—Sm1—C3—C2	−105.04 (10)	C15—N2—C28—C33	−114.88 (19)
C2—C3—C4—C5	0.1 (4)	Sm1—N2—C28—C33	101.8 (2)
Sm1—C3—C4—C5	45.4 (2)	C33—C28—C29—C30	1.8 (3)
C2—C3—C4—Sm1	−45.3 (2)	N2—C28—C29—C30	−179.67 (17)
N2—Sm1—C4—C3	−54.00 (12)	C33—C28—C29—C34	−177.38 (17)
O1L—Sm1—C4—C3	146.16 (12)	N2—C28—C29—C34	1.1 (3)
N1—Sm1—C4—C3	−109.91 (11)	C28—C29—C30—C31	−0.6 (3)
C2—Sm1—C4—C3	18.27 (10)	C34—C29—C30—C31	178.6 (2)
C8—Sm1—C4—C3	73.64 (11)	C29—C30—C31—C32	−1.1 (3)
C7—Sm1—C4—C3	103.88 (11)	C30—C31—C32—C33	1.7 (4)
C1—Sm1—C4—C3	42.75 (10)	C31—C32—C33—C28	−0.5 (3)
C5—Sm1—C4—C3	149.18 (17)	C31—C32—C33—C37	176.2 (2)
C6—Sm1—C4—C3	129.90 (12)	C29—C28—C33—C32	−1.3 (3)
C15—Sm1—C4—C3	−82.41 (11)	N2—C28—C33—C32	−179.86 (17)
N2—Sm1—C4—C5	156.81 (10)	C29—C28—C33—C37	−177.94 (18)
O1L—Sm1—C4—C5	−3.02 (18)	N2—C28—C33—C37	3.5 (3)
N1—Sm1—C4—C5	100.91 (11)	C30—C29—C34—C36	−47.3 (2)
C2—Sm1—C4—C5	−130.91 (12)	C28—C29—C34—C36	131.83 (19)
C8—Sm1—C4—C5	−75.54 (11)	C30—C29—C34—C35	75.8 (2)
C7—Sm1—C4—C5	−45.31 (11)	C28—C29—C34—C35	−105.1 (2)
C1—Sm1—C4—C5	−106.44 (11)	C32—C33—C37—C39	32.4 (3)
C3—Sm1—C4—C5	−149.18 (17)	C28—C33—C37—C39	−150.99 (19)
C6—Sm1—C4—C5	−19.28 (10)	C32—C33—C37—C38	−90.5 (2)
C15—Sm1—C4—C5	128.40 (11)	C28—C33—C37—C38	86.1 (2)
C3—C4—C5—C6	4.0 (4)	C10L—C5L—C6L—C7L	0.0
Sm1—C4—C5—C6	49.5 (2)	C11L—C5L—C6L—C7L	−178.0 (3)
C3—C4—C5—Sm1	−45.5 (2)	C5L—C6L—C7L—C8L	0.0
N2—Sm1—C5—C4	−37.34 (16)	C6L—C7L—C8L—C9L	0.0
O1L—Sm1—C5—C4	178.40 (10)	C7L—C8L—C9L—C10L	0.0
N1—Sm1—C5—C4	−87.03 (11)	C8L—C9L—C10L—C5L	0.0
C2—Sm1—C5—C4	41.09 (11)	C6L—C5L—C10L—C9L	0.0
C8—Sm1—C5—C4	101.15 (11)	C11L—C5L—C10L—C9L	178.1 (3)
C7—Sm1—C5—C4	125.30 (13)	C46—C41—C42—C43	0.0
C1—Sm1—C5—C4	71.26 (11)	C47—C41—C42—C43	178.6 (16)
C3—Sm1—C5—C4	17.79 (10)	C41—C42—C43—C44	0.0
C6—Sm1—C5—C4	146.70 (17)	C42—C43—C44—C45	0.0
C15—Sm1—C5—C4	−65.73 (12)	C43—C44—C45—C46	0.0
N2—Sm1—C5—C6	175.96 (9)	C44—C45—C46—C41	0.0
O1L—Sm1—C5—C6	31.71 (13)	C42—C41—C46—C45	0.0
N1—Sm1—C5—C6	126.27 (10)	C47—C41—C46—C45	−178.6 (16)
